# *ADAM10* as a Potential Biomarker in Non-Small Cell Lung Cancer and Its Major Histologic Subtypes

**DOI:** 10.3390/medicina62102015

**Published:** 2026-10-18

**Authors:** Sae-Beaul Kim, Yun-Jeong Kim, Yunha Lee, Ji-Hun An, Min-Gyeol Kim, Jin Lee, Ye-Rin Jung, Jun-Chae Lee, Jae-Ho Lee, Taehun Kim

**Affiliations:** 1 Medical Course, School of Medicine, Keimyung University, Daegu 42601, Republic of Korea; hakumc2014@gmail.com (S.-B.K.); yunjeong20060808@gmail.com (Y.-J.K.); lyh3dland3@gmail.com (Y.L.); jhahn2747@gmail.com (J.-H.A.); rlaalsruf0914@gmail.com (M.-G.K.); okm333o@naver.com (J.L.); camexxia@naver.com (Y.-R.J.); chai1703@naver.com (J.-C.L.); 2 Department of Anatomy, School of Medicine, Keimyung University, Daegu 42601, Republic of Korea; anato82@dsmc.or.kr; 3 Division of Pulmonary Medicine, Department of Internal Medicine, Keimyung University Dongsan Hospital, Keimyung University School of Medicine, Daegu 42601, Republic of Korea

**Keywords:** *ADAM10*, non-small cell lung cancer, lung adenocarcinoma, lung squamous cell carcinoma, prognosis, gene expression, TCGA

## Abstract

*Background and Objectives*: A disintegrin and metalloproteinase 10 (*ADAM10*) is a membrane-anchored metalloprotease involved in ectodomain shedding and cancer progression. However, its molecular and prognostic significance across major histological subtypes of non-small cell lung cancer (NSCLC) remains unclear. This study evaluated the clinical and molecular relevance of *ADAM10* in NSCLC, focusing on lung adenocarcinoma (LUAD) and lung squamous cell carcinoma (LUSC). *Materials and Methods*: Transcriptomic and clinicopathological data from 980 patients with NSCLC, including 491 patients with LUAD and 489 patients with LUSC, were obtained from The Cancer Genome Atlas through the OncoLnc database. Patients in each cohort were classified into *ADAM10*-high and *ADAM10*-low groups according to the median *ADAM10* mRNA expression level. Univariate and multivariate Cox proportional hazards regression analyses were performed to evaluate the prognostic significance of *ADAM10* expression. *Results*: *ADAM10* expression was not significantly associated with baseline clinicopathological characteristics in the overall cohort or within the LUAD and LUSC subgroups. However, *ADAM10* expression showed distinct molecular associations according to histologic subtype. In LUAD, *ADAM10* expression was positively correlated with *KRAS* expression (ρ = 0.286, *p* < 0.001), *BRAF* expression (ρ = 0.145, *p* = 0.001), and *EGFR* expression (ρ = 0.187, *p* < 0.001), while it was negatively correlated with *TP53* expression (ρ = −0.12, *p* = 0.005), whereas in LUSC, it was positively correlated with *PIK3CA*. High *ADAM10* expression was associated with significantly poorer EFS and OS in the overall NSCLC cohort. In multivariate analyses, high *ADAM10* expression remained an independent predictor of shorter EFS (HR = 1.352, 95% CI = 1.041–1.755, *p* = 0.024) and OS (HR = 1.335, 95% CI = 1.089–1.636, *p* = 0.005), independent of clinicopathological factors. *Conclusions*: High *ADAM10* expression was independently associated with poorer survival outcomes in NSCLC, with a more pronounced prognostic impact in LUAD than in LUSC. These findings suggest that *ADAM10* may serve as a potential prognostic biomarker in NSCLC, particularly in LUAD.

## 1. Introduction

Lung cancer remains one of the most frequently diagnosed malignancies and the leading cause of cancer-related mortality worldwide [1,2]. Its development and progression are characterized by substantial biological heterogeneity, with distinct histologic and molecular subtypes, including tumors harboring different oncogenic driver alterations, contributing to differences in tumor behavior and treatment response [3,4]. Histologically, lung cancer is broadly classified into small cell lung cancer and non-small cell lung cancer (NSCLC), with NSCLC accounting for approximately 85% of lung cancer cases [5]. Lung adenocarcinoma (LUAD) and lung squamous cell carcinoma (LUSC) represent the two major histological subtypes of NSCLC and exhibit distinct molecular and clinical characteristics [5,6]. These differences provide a rationale for evaluating molecular factors according to histological subtype, as the identification of actionable molecular alterations increasingly guides the selection of standard targeted therapies and systemic treatment strategies in NSCLC [7,8].

A disintegrin and metalloproteinase 10 (*ADAM10*) is a membrane-anchored metalloprotease of the ADAM family that mediates the ectodomain shedding of multiple cell-surface proteins [9,10]. *ADAM10* functions as a major α-secretase and regulates cell-surface signaling by cleaving and releasing the ectodomains of various membrane proteins [9,10]. In the lung, *ADAM10* has been implicated in fibrotic remodeling as an important regulator of fibroblast activation through proteolytic shedding of cell-surface proteins. Given that its substrates also participate in cell–cell interactions and signal transduction, alterations in *ADAM10* activity have also been implicated in tumor progression, including enhanced migration and invasion in non-small cell lung cancer [11,12,13].

Evidence from NSCLC studies supports a potential association between *ADAM10* and tumor progression. Guo et al. reported increased *ADAM10* expression in NSCLC tissues and showed that inhibition of *ADAM10* reduced the migration and invasion of lung cancer cells, with the Notch1 signaling pathway implicated in these effects [12]. In addition, Yoneyama et al. demonstrated increased *ADAM10*-specific sheddase activity in NSCLC cell lines and primary tumors and reported an association between higher *ADAM10* activity and clinical outcome [14]. More recently, Zhang et al. reported that *ADAM10* expression was associated with overall and recurrence-free survival in NSCLC and that experimental modulation of *ADAM10* affected malignant cellular phenotypes [13]. However, previous studies have primarily evaluated the prognostic association of *ADAM10* using unadjusted survival analyses, with limited assessment of clinically relevant oncogenic driver alterations or other potential confounders. In particular, whether the prognostic significance of *ADAM10* expression persists after accounting for tumor stage and molecular characteristics remains unclear.

LUAD and LUSC differ substantially in their genomic alterations and signaling environments, including differences in frequently altered driver genes and therapeutic targets [15]. Accordingly, we analyzed transcriptomic and clinicopathological data from 980 patients with NSCLC in The Cancer Genome Atlas (TCGA), including 492 patients with LUAD and 488 with LUSC. We evaluated the associations of *ADAM10* expression with clinicopathological characteristics, clinically relevant oncogenic driver alterations, including EGFR and KRAS, and overall survival (OS) and recurrence-free survival (RFS) using stage-adjusted analyses. By analyzing LUAD and LUSC separately, we aimed to characterize the subtype-specific clinical, molecular, and prognostic significance of *ADAM10* expression.

## 2. Materials and Methods

### 2.1. TCGA Data Acquisition and Patient Selection

**Figure 1:**
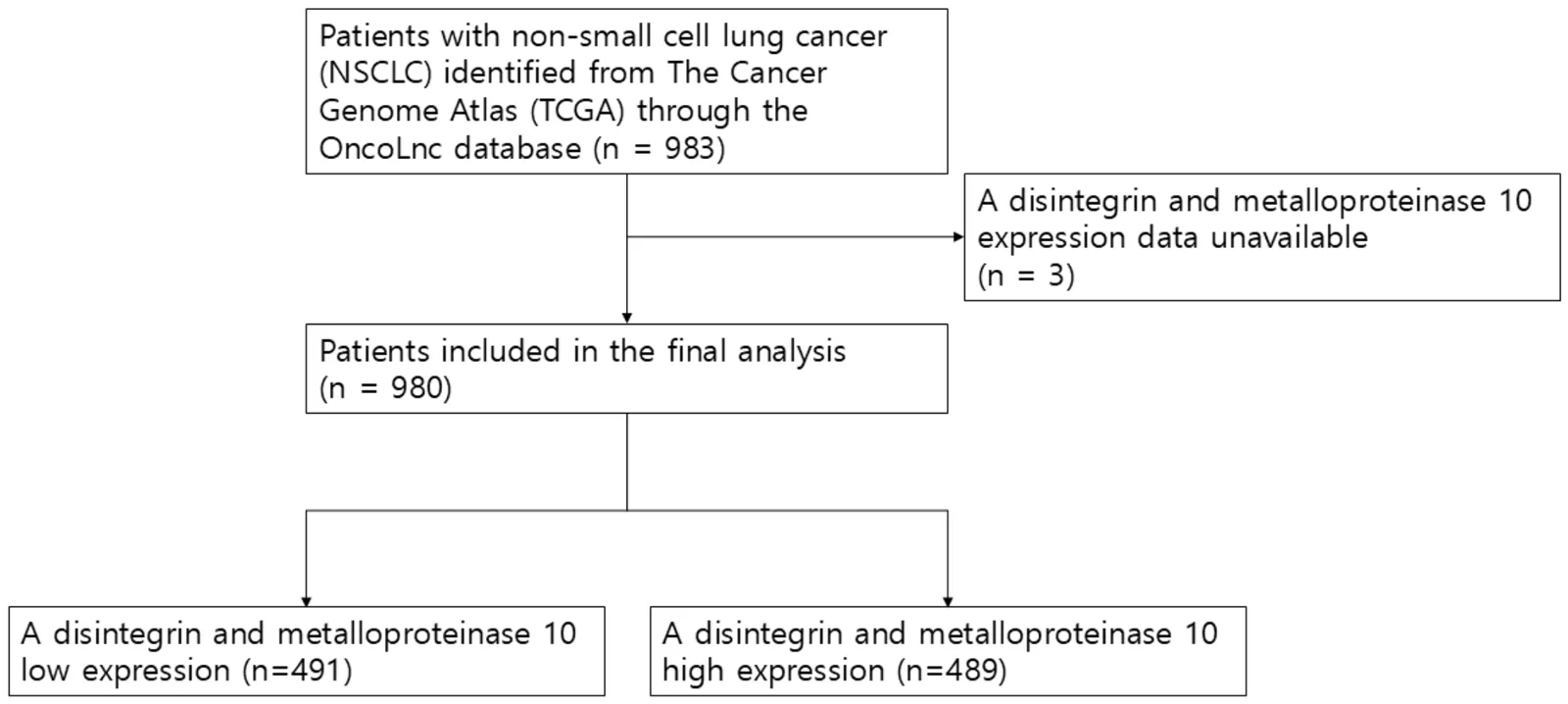
Flow diagram of the study population and analysis.

We utilized primary data from The Cancer Genome Atlas (TCGA) portal (http://cancergenome.nih.gov/) (accessed on 14 October 2025). An initial pan-cancer survival screening was performed to evaluate the prognostic relevance of *ADAM10* expression across multiple human malignancies. Patients with LUAD and LUSC were included in the present study. Among 983 initially identified patients, three patients without available *ADAM10* expression data were excluded, resulting in a final cohort of 980 patients, comprising 492 with LUAD and 488 with LUSC. *ADAM10* mRNA expression and clinicopathological data, including age, sex, smoking status, pathologic stage, tumor–node–metastasis classification, and EGFR mutation status, were collected when available (Figure 1).

Patients were classified into *ADAM10*-high and *ADAM10*-low groups according to the median *ADAM10* mRNA expression level in the overall cohort, with 491, and 489 patients in each group respectively. Clinicopathological characteristics, molecular features, and survival outcomes were compared between the *ADAM10*-high and *ADAM10*-low groups. These analyses were further performed separately in LUAD and LUSC to assess histological subtype-specific associations. Patients with missing information for a specific variable were excluded only from the corresponding analysis.

### 2.2. Data Collection

Clinical, pathological, molecular, and outcome data were collected from the NSCLC cohort. Demographic and clinical characteristics, including age, sex, and smoking history, were collected. Pathological data included histological subtype (LUAD vs. LUSC) and pathologic TNM stage. Pathologic TNM stage was determined according to the 8th edition of the American Joint Committee on Cancer (AJCC) TNM classification for lung cancer [16]. The stage was dichotomized into early-stage disease, comprising stages I–II, and advanced-stage disease, comprising stages III–IV. *ADAM10* expression status was also assessed. *ADAM10* mRNA expression data were obtained from TCGA through the OncoLnc database and are presented as normalized RSEM expression values. Molecular alterations, including *EGFR*, *ALK*, *KRAS*, *BRAF*, and *TP53*, were collected for the overall NSCLC cohort, while *TP53*, *CDKN2A*, *SOX2*, *PIK3CA*, and *NOTCH1* alterations were additionally evaluated in the LUSC cohort. Outcome data included OS, event-free survival (EFS), and the corresponding follow-up durations. Patients without recurrence information were excluded from the EFS analysis. Patients who did not experience the event of interest were censored at the last follow-up. EFS was defined as the time from diagnosis to the first occurrence of disease progression, recurrence, or death from any cause. The median follow-up duration was estimated by the reverse Kaplan-Meier method, and the number of events in each group was determined for each histologic subtype and *ADAM10* expression group. Clinical testing identified mutations in EGFR (*n* = 29), KRAS (*n* = 64), MET (*n* = 15), ERBB2 (*n* = 6), and BRAF (*n* = 18). Due to the small sample sizes in the other mutation groups, statistical comparisons of *ADAM10* expression were limited to the EGFR, KRAS, and BRAF mutation groups.

### 2.3. Statistical Analysis

Categorical variables were compared between the *ADAM10*-high and *ADAM10*-low groups using the chi-square test or Fisher’s exact test, as appropriate. Pearson correlation analysis was performed to evaluate the relationships between *ADAM10* mRNA expression and continuous clinical variables or selected lung cancer-related genes.

The continuous clinical variables included age and the number of pack-years smoked. In the LUAD cohort, *ADAM10* expression was correlated with the expression levels of *KRAS*, *TP53*, *ALK*, *BRAF*, and *EGFR*. In the LUSC cohort, *ADAM10* expression was correlated with *TP53*, *CDKN2A*, *SOX2*, *PIK3CA*, and *NOTCH1*.

Associations between variables were assessed using Spearman’s rank correlation analysis. Spearman correlation coefficients (r) were reported, with positive r values indicating direct correlations and negative r values indicating inverse correlations. The prognostic significance of *ADAM10* expression was evaluated using OS and EFS. Kaplan–Meier survival curves were generated separately for the *ADAM10*-high and *ADAM10*-low groups in the LUAD and LUSC cohorts. Differences between the survival curves were evaluated using the log-rank test. The chi-square statistics and corresponding *p*-values were reported for each survival analysis. Univariate and multivariate Cox proportional hazards regression analyses were performed to determine whether *ADAM10* expression was independently associated with OS. The variables included in the analyses were age, gender, smoking status, pathologic stage, EGFR mutation status, and *ADAM10* expression group. Age was analyzed as >65 versus ≤65 years, pathologic stage as stages III–IV versus stages I–II, EGFR mutation status as mutant versus wild type, and *ADAM10* expression as high versus low. To assess whether the association between *ADAM10* expression and survival differed according to histological subtype, an interaction term between *ADAM10* expression and histological subtype (*ADAM10* × histology) was included in the multivariable model. *ADAM10* expression was analyzed as a continuous variable after standardization to a z-score, and hazard ratios (HRs) with 95% confidence intervals (CIs) were reported per 1-standard-deviation increase in *ADAM10* expression. Hazard ratios (HRs) and corresponding 95% confidence intervals (CIs) were calculated. Forest plots were constructed to visualize the results of the Cox regression analyses. All statistical tests were two-sided. A *p*-value <0.05 was considered statistically significant, whereas *p*-values between 0.05 and 0.10 were described as borderline. All statistical analyses were performed using IBM SPSS Statistics, version 32.0 (IBM Corp., Armonk, NY, USA).

## 3. Results

### 3.1. Baseline Characteristics and ADAM10 Expression

In the overall NSCLC cohort, *ADAM10* expression was significantly associated with age, gender, and histologic diagnosis. The high-expression group included more patients aged >65 years and fewer females, while LUAD was more frequent in the low-expression group. Smoking status did not differ significantly between the groups (Table 1). The associations between *ADAM10* expression and clinicopathological characteristics were also evaluated separately in the LUAD and LUSC cohorts respectively (Supplementary Table S1). In the LUAD and LUSC cohorts, respectively, no statistically significant associations were observed between *ADAM10* expression and age, gender, smoking status, or pathologic stage. We further evaluated the correlations between *ADAM10* expression and clinical or molecular variables in the LUAD and LUSC cohorts (Supplementary Tables S2 and S3). In the LUAD cohort, *ADAM10* expression was positively correlated with *KRAS* expression (ρ = 0.286, *p* < 0.001), *BRAF* expression (ρ = 0.145, *p* = 0.001), and *EGFR* expression (ρ = 0.187, *p* < 0.001), while it was negatively correlated with *TP53* expression (ρ = −0.12, *p* = 0.005). No other statistically significant correlations were observed. In the LUSC cohort, *ADAM10* expression was positively correlated with *PIK3CA* expression (ρ = 0.269, *p* < 0.001) and age (ρ = 0.122, *p* = 0.007). No other statistically significant correlations were identified. *ADAM10* expression did not differ significantly between mutant and wild-type tumors for either EGFR (median [IQR], 1523.99 [1221.28–1944.91] vs. 1657.82 [1196.67–2158.29]; *p* = 0.438) or KRAS (1640.51 [1194.10–2180.25] vs. 1657.68 [1198.66–2095.51]; *p* = 0.986; Mann–Whitney U test) (Supplementary Table S4).

### 3.2. Survival Analysis According to ADAM10 Expressions

**Figure 2:**
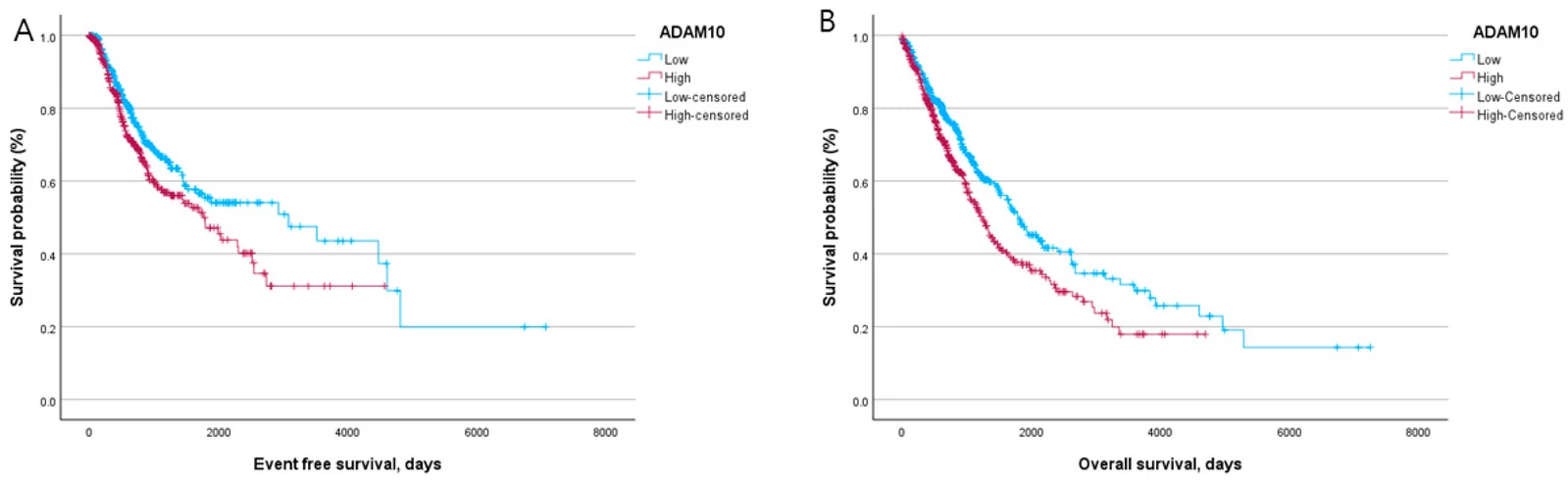
Kaplan–Meier survival curves according to *ADAM10* expression in patients with NSCLC. (**A**) Event-free survival (EFS) according to *ADAM10* expression. (*ADAM10*-low, *n* = 385; *ADAM10*-high, *n* = 409; log-rank *p* = 0.015). (**B**) Overall survival (OS) according to *ADAM10* expression. (*ADAM10*-low, *n* = 488; *ADAM10*-high, *n* = 489; log-rank *p* = 0.001). Patients were classified into *ADAM10*-high and *ADAM10*-low groups according to the median *ADAM10* mRNA expression (normalized RSEM values) in the overall cohort. Patients without the event were censored at the last follow-up (tick marks). Survival curves were estimated by the Kaplan-Meier method and compared using the log-rank test.

Kaplan–Meier survival analyses were performed according to *ADAM10* expression levels in the participants. Both EFS and OS were significantly worse in the *ADAM10* high-expression group than in the low-expression group (Figure 2A,B). Among 794 patients, 243 (30.6%) experienced EFS events. Median EFS in the overall cohort was 5.4 years (95% CI, 4.0–6.9). EFS events occurred in 109/385 patients (28.3%) in the *ADAM10*-low group and 134/409 (32.8%) in the *ADAM10*-high group. Median EFS was 8.4 years (95% CI, 4.0–12.9) and 4.9 years (95% CI, 3.7–6.1), respectively (log-rank *p* = 0.015). OS events occurred in 177/488 patients (36.3%) in the *ADAM10*-low group and 213/489 (43.6%) in the *ADAM10*-high group. Median OS was 4.9 years (95% CI, 4.2–5.7) and 3.4 years (95% CI, 2.9–3.9), respectively (log-rank *p* = 0.001).

### 3.3. Cox Proportional Hazards Regression Analysis

Univariate and multivariate Cox proportional hazards regression analyses were performed to identify factors associated with EFS and OS. For EFS, advanced pathologic stage (stage III–IV vs. I–II; multivariate HR = 1.702, 95% CI = 1.257–2.305, *p* < 0.001), LUAD histology (vs. LUSC; HR = 1.748, 95% CI = 1.310–2.310, *p* < 0.001), and high *ADAM10* expression (HR = 1.414, 95% CI = 1.082–1.848, *p* = 0.011) were independently associated with shorter EFS. Age, sex, and smoking status were not significant independent predictors of EFS (Table 2). For OS, advanced pathologic stage remained an independent predictor of poorer survival (HR = 1.847, 95% CI = 1.476–2.313, *p* < 0.001). High *ADAM10* expression was also independently associated with shorter OS (HR = 1.373, 95% CI = 1.109–1.699, *p* = 0.004) (Table 3). When *ADAM10* expression was analyzed as a continuous standardized variable, each 1-SD increase in *ADAM10* expression was independently associated with a higher risk of death (adjusted HR, 1.157; 95% CI, 1.053–1.271; *p* = 0.002) and a higher risk of an event (adjusted HR, 1.252; 95% CI, 1.109–1.413; *p* < 0.001). Age showed a borderline association with OS in the multivariate analysis (HR = 1.229, 95% CI = 0.999–1.513, *p* = 0.051), whereas sex, smoking status, and histologic subtype were not significant independent predictors of OS.

### 3.4. Histology-Specific Analyses

To examine whether the prognostic association of *ADAM10* differed by histology, we first tested the interaction between *ADAM10* expression and histologic subtype in the Cox model. The interaction was not statistically significant for either EFS (*p* for interaction = 0.186) or OS (*p* for interaction = 0.684) (Table 2 and Table 3). Nevertheless, given the biological and clinical differences between LUAD and LUSC, each subtype was also analyzed separately. Among patients with LUSC, EFS events occurred in 99/379 (26.1%) and deaths in 212/487 (43.5%); among those with LUAD, EFS events occurred in 144/415 (34.7%) and deaths in 178/490 (36.3%). Median EFS was 6.3 years (95% CI, 3.9–8.8) in LUSC and 3.8 years (95% CI, 1.6–5.9) in LUAD. Median OS was 4.5 years (95% CI, 3.5–5.5) in LUSC and 4.0 years (95% CI, 3.4–4.6) in LUAD. In the LUAD cohort, both OS and EFS were significantly shorter in the *ADAM10*-high group than in the *ADAM10*-low group (Supplementary Figure S1A,B and Table 4). In the LUSC cohort, OS showed a borderline difference between the two groups, whereas no significant difference was observed in EFS (Supplementary Figure S2A,B and Table 4). When the analyses were performed separately by histologic subtype, advanced pathologic stage remained independently associated with poorer OS in both the LUAD and LUSC cohorts (Supplementary Tables S5 and S6). The association between high *ADAM10* expression and OS was not significant after multivariate adjustment in either cohort.

## 4. Discussion

In this TCGA-based analysis of NSCLC, higher *ADAM10* mRNA expression was associated with shorter EFS and OS. The association remained significant after adjustment for stage and other clinical covariates, both with the median-based dichotomization and when *ADAM10* was modeled as a continuous standardized variable (adjusted HR per 1-SD increase, 1.157 for OS and 1.252 for EFS). The consistency of the direction and magnitude across these approaches suggests that the finding is not merely an artifact of the chosen cutoff. These results are in line with previous reports linking *ADAM10* to malignant phenotypes and adverse outcomes in NSCLC [12,13,14]. Zhang et al. reported that high *ADAM10* expression was associated with shorter overall and recurrence-free survival using the Kaplan-Meier Plotter and a meta-analysis of public datasets, together with functional experiments in lung cancer cell lines [13]. Our study adds multivariable Cox analyses using patient-level TCGA data, a continuous-variable analysis, and a formal test of interaction with histologic subtype, but the main prognostic finding should be regarded as confirmatory.

*ADAM10* is a membrane-bound sheddase that cleaves a broad range of substrates, including Notch receptors, E-cadherin, EGFR/HER ligands, and PD-L1 [17]. Through these substrates, it can plausibly influence proliferation, invasion, and immune evasion. In NSCLC, *ADAM10* overexpression has been linked to Notch1 activation and to cell migration and invasion [12], and an active form of *ADAM10* has been reported to mark cancer stem-like cells with high Notch activity [18]. In LUAD, *ADAM10* expression was positively correlated with *KRAS*, *BRAF*, and *EGFR* expression and negatively correlated with *TP53* expression, which is compatible with involvement of the EGFR-RAS-MAPK axis, given that *ADAM10* releases EGFR/HER ligands [17]. In LUSC, *ADAM10* was positively correlated with *PIK3CA*, a gene frequently amplified in this subtype. However, these correlations were weak (|r| = 0.09–0.29, explaining less than 10% of the variance) and were obtained from mRNA levels. Moreover, *ADAM10* expression did not differ between *EGFR* or *KRAS* mutant and wild-type tumors. These observations indicate that the relationship reflects expression-level co-variation rather than mutation-driven regulation, and they should be interpreted as hypothesis-generating rather than as evidence of a mechanistic link.

With regard to histology, the association between high *ADAM10* expression and survival was evident in LUAD in the stratified Kaplan-Meier analyses, whereas in LUSC it was borderline for OS and absent for EFS. Nevertheless, the interaction between *ADAM10* expression and histologic subtype was not significant for either EFS (*p* for interaction = 0.186) or OS (*p* = 0.684), and the association was not significant after multivariable adjustment within either subtype. We therefore cannot conclude that the prognostic effect differs by histology. The subtype-specific analyses have fewer events and lower statistical power, so the loss of significance may reflect limited power rather than true absence of an effect. Conversely, the non-significant interaction test does not prove that the effect is identical across subtypes. We do not claim that *ADAM10* is a subtype-specific independent prognostic biomarker.

This study has several limitations. First, and most importantly, our findings were not validated in an independent external cohort. All analyses were performed within a single public dataset (TCGA), and the prognostic association of *ADAM10* expression, the cutoff-independent results, and the subtype-specific findings could therefore reflect cohort-specific characteristics, including sample selection, platform, and batch effects, and may not be generalizable to other populations or expression platforms. Validation in independent cohorts, such as GEO datasets or prospective clinical series, is needed before any prognostic role can be established. Second, this was a retrospective analysis of a public database, and information on treatment, tumor purity, and other potential confounders was limited. Third, only mRNA expression was assessed, and ADAM10 protein level and proteolytic activity, which are more directly relevant to its function and have been proposed as biomarkers in NSCLC [14], were not evaluated. Fourth, the median cutoff is arbitrary, although the continuous analysis supported the main findings. Fifth, the subtype-specific analyses included fewer events and had limited statistical power, and the EFS analysis included fewer patients (*n* = 794) than the OS analysis (*n* = 977) because of missing data. Finally, multiple correlations were examined, so the weak associations may include false-positive findings, and the effect sizes were modest. The clinical utility of *ADAM10* expression as a stand-alone biomarker therefore remains to be established.

## 5. Conclusions

High *ADAM10* expression was associated with worse EFS and OS in NSCLC, independent of pathologic stage, and this association was consistent when *ADAM10* was analyzed as a continuous variable. The prognostic association was more evident in LUAD in stratified analyses, but no significant interaction with histology was observed, and the subtype-specific multivariable analyses did not reach significance. These findings support *ADAM10* as a candidate prognostic marker in NSCLC and warrant validation in independent cohorts with protein-level and functional data.

## Figures and Tables

**Table 1:** Clinicopathological characteristics according to *ADAM10* expression in NSCLC.

	NSCLC (*n* = 980)		
	*ADAM10* High Group (*n* = 491)	*ADAM10* Low Group (*n* = 489)	*p*-Value
Age			
>65	302 (61.6%)	250 (51.3%)	0.001
Gender			
Female	166 (33.8%)	225 (46.0%)	<0.001
Smoking Status			
Ever smoker	378 (77.0%)	368 (75.3%)	0.525
Never smoker	113 (23.0%)	121 (24.7%)	
Diagnosis			
LUAD	185 (37.7%)	307 (62.8%)	<0.001
*ADAM10* mRNA expression (normalized RSEM)	2241.34 (1943.89–2814.95)	1251.43 (1006.50–1515.80)	<0.001
Pathologic stage			
Stage I	237 (48.3%)	278 (56.9%)	0.056
Stage II	151 (30.8%)	121 (24.7%)	
Stage III	88 (17.9%)	74 (15.1%)	
Stage IV	15 (3.1%)	16 (3.3%)	

Data are presented as mean ± standard deviation (SD), median (IQR), or *n* (%). *ADAM10*, a disintegrin and metalloproteinase 10; NSCLC, non-small cell lung cancer; LUAD, lung adenocarcinoma; *ADAM10* expression is presented as normalized RSEM values (TCGA, via OncoLnc), median (IQR). High and low groups were defined by the overall cohort median.

**Table 2:** Univariate and multivariate Cox proportional hazards regression analyses of event free survival in patients with NSCLC.

Variable	Univariate HR (95% CI)	*p* Value	Multivariate HR (95% CI)	*p* Value
Age (>65 vs. ≤65)	1.125 (0.871–1.452)	0.368	1.209 (0.931–1.571)	0.155
Gender (Male vs. Female)	1.031 (0.800–1.330)	0.812	1.077 (0.825–1.077)	0.587
Smoking (Yes vs. No)	0.958 (0.716–1.283)	0.774	1.077 (0.794–1.460)	0.633
Stage (III–IV vs. I–II)	1.776 (1.315–2.399)	<0.001	1.702 (1.257–2.305)	<0.001
Pathology (LUAD vs. LUSC)	1.507 (1.164–1.951)	0.002	1.748 (1.310–2.310)	<0.001
*ADAM10* (High vs. Low)	1.260 (0.978–1.623)	0.074	1.414 (1.082–1.848)	0.011
*ADAM10* × Pathology	—	—	1.425 (0.843–2.410)	0.186

NSCLC, non-small cell lung cancer; HR, hazard ratio; CI, confidence interval; LUAD, lung adenocarcinoma; LUSC, lung squamous cell carcinoma.

**Table 3:** Univariate and multivariate Cox proportional hazards regression analyses of overall survival in patients with NSCLC.

Variable	Univariate HR (95% CI)	*p* Value	Multivariate HR (95% CI)	*p* Value
Age (>65 vs. ≤65)	1.251 (1.020–1.534)	0.032	1.229 (0.999–1.512)	0.051
Gender (Male vs. Female)	1.080 (0.975–1.198)	0.143	1.100 (0.886–1.365)	0.388
Smoking (Yes vs. No)	0.944 (0.745–1.197)	0.636	0.911 (0.712–1.167)	0.460
Stage (III-IV vs. II-II)	1.885 (1.509–2.356)	<0.001	1.847 (1.476–2.313)	<0.001
Pathology (LUAD vs. LUSC)	0.907 (0.742–1.109)	0.340	0.825 (0.819–1.284)	0.825
*ADAM10* (High vs. Low)	1.448 (1.184–1.770)	<0.001	1.373 (1.109–1.699)	0.004
*ADAM10* × Pathology	—	—	1.088 (0.725–1.633)	0.684

NSCLC, non-small cell lung cancer; HR, hazard ratio; CI, confidence interval; LUAD, lung adenocarcinoma; LUSC, lung squamous cell carcinoma.

**Table 4:** Kaplan–Meier log-rank test results according to ADAM10 expression.

Survival Type	Cancer Type	Chi-Square	*p*-Value
OS	LUAD	5.937	0.015
EFS	LUAD	6.017	0.014
OS	LUSC	3.774	0.052
EFS	LUSC	0.158	0.691

Survival curves were compared between the ADAM10-high and ADAM10-low expression groups using the Kaplan–Meier method and log-rank test. Chi-square statistics and corresponding *p* values are presented. OS, overall survival; EFS, event-free survival; LUAD, lung adenocarcinoma; LUSC, lung squamous cell carcinoma.

## Data Availability

Publicly available datasets were analyzed in this study. These data can be found in The Cancer Genome Atlas (TCGA) database (https://portal.gdc.cancer.gov/, accessed on accessed on 13 August 2026).

## Supplementary Materials

The following supporting information can be downloaded at https://www.mdpi.com/article/10.3390/medicina62102015/s1, Figure S1: Kaplan–Meier curves for overall survival (A) and recurrence-free survival (B) according to *ADAM10* expression in LUAD. Figure S2: Kaplan–Meier curves for overall survival (A) and recurrence-free survival (B) according to *ADAM10* expression in LUSC. Supplement Table S1. Clinicopathological characteristics according to *ADAM10* expression in LUAD and LUSC. Table S2: Pearson correlation analysis in the LUAD cohort. Table S3: Pearson correlation analysis in the LUSC cohort. Table S4: Comparison of *ADAM10* expression according to KRAS and EGFR mutation status. Table S5: Univariate and multivariate Cox proportional hazards regression analyses of overall survival in patients with LUAD. Table S6: Univariate and multivariate Cox proportional hazards regression analyses of overall survival in patients with LUSC.
