# Supplementary material for: ADAM10 as a Potential Biomarker in Non-Small Cell Lung Cancer and Its Major Histologic Subtypes
Source: Medicina (Kaunas). 2026 Oct 18;62(10):2015. doi: 10.3390/medicina62102015 (PMC13706909; doi:10.3390/medicina62102015)
Supplement: Supplementary file 1 [file medicina-62-02015-s001.zip › medicina-4633344-supplementary.pdf]

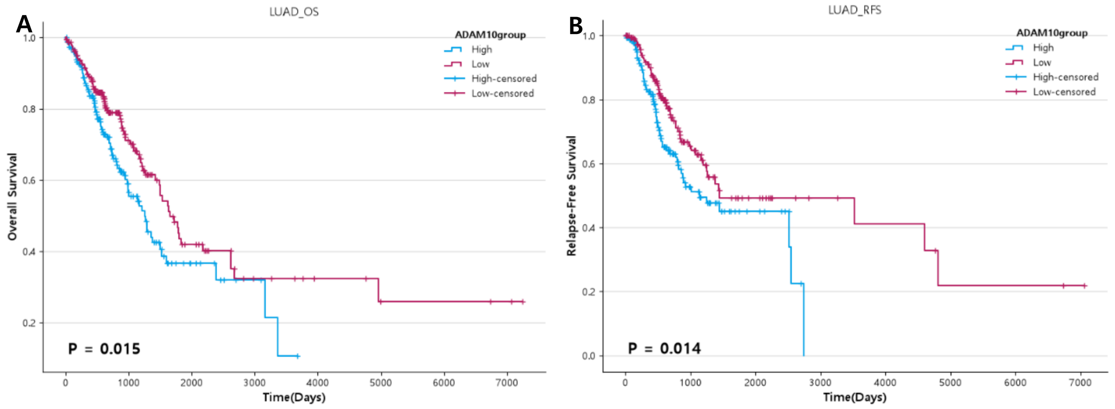

Supplementary Figure S1. Kaplan–Meier curves for overall survival (A) and recurrence-free survival (B) according to ADAM10 expression in LUAD.

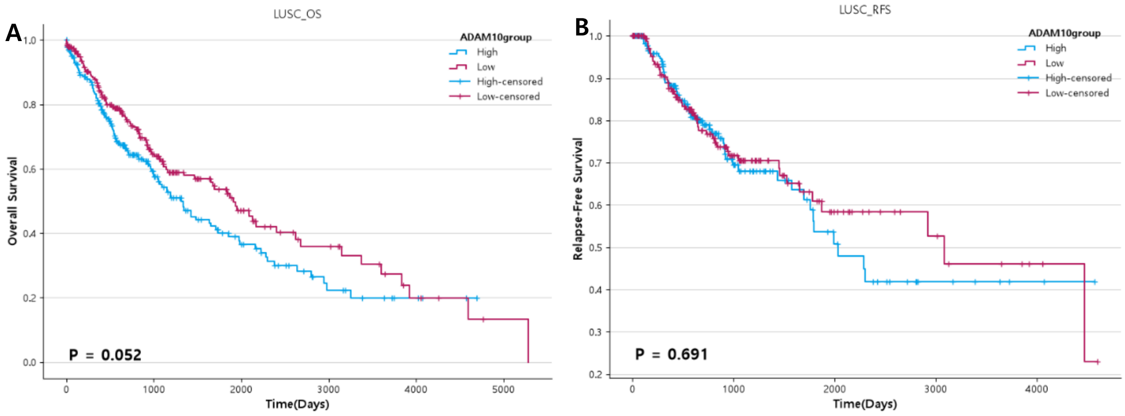

Supplementary Figure S2. Kaplan–Meier curves for overall survival (A) and recurrence-free survival (B) according to ADAM10 expression in LUSC.

Supplement Table S1. Clinicopathological characteristics according to ADAM10 expression in LUAD and LUSC.

	LUAD (N=492)			LUSC (N=488)		
	High	Low	P-value	High 244	Low 244	P-value
**Age**						
≤65	111	125	0.206	86	103	0.106
>65	135	121		158	141	
**Gender**						
Female	125	141	0.349	61	64	0.735
Male	120	104		183	180	
**Smoking Status**						
Ever	164	169	0.630	204	209	0.530
Never	82	77		40	35	
**Pathologic stage**						
Stage I	123	149	0.071	121	122	0.955
Stage II	62	55		79	76	
Stage III	46	33		40	43	
Stage IV	15	9		4	3	

Data are presented as n. ADAM10, a disintegrin and metalloproteinase 10; NSCLC, non-small cell lung cancer; LUAD, lung adenocarcinoma;

Supplementary Table S2. Spearman correlation analysis in the LUAD cohort.

		ADAM10	Pack-Years	Age	KRAS	TP53	ALK	BRAF	EGFR
ADAM10	ρ	1	.031	.075	.286**	-.127**	.006	.145**	.187**
	P		.570	.095	<.001	.005	.890	.001	<.001
Pack-Years	ρ	.031	1	.052	-.074	.013	-.052	.032	-.037
	P	.570		.344	.180	.815	.346	.566	.498
Age	ρ	.075	.052	1	.049	.064	.010	-.043	.015
	P	.095	.344		.275	.156	.817	.344	.744
KRAS	ρ	.286**	-.074	.049	1	-.030	-.089*	.188**	.012
	P	<.001	.180	.275		.507	.049	<.001	.790
TP53	ρ	-.127**	.013	.064	-.030	1	.026	-.035	-.102*
	P	.005	.815	.156	.507		.563	.435	.024
ALK	ρ	.006	-.052	.010	-.089*	.026	1	-.098*	.065
	P	.890	.346	.817	.049	.563		.030	.153
BRAF	ρ	.145**	.032	-.043	.188**	-.035	-.098*	1	.298**
	P	.001	.566	.344	<.001	.435	.030		<.001
EGFR	ρ	.187**	-.037	.015	.012	-.102*	.065	.298**	1
	P	<.001	.498	.744	.790	.024	.153	<.001	

LUAD, lung adenocarcinoma; ADAM10, a disintegrin and metalloproteinase 10; KRAS, Kirsten rat sarcoma viral oncogene; TP53, tumor protein p53; ALK, anaplastic lymphoma kinase; BRAF, B-Raf proto-oncogene; EGFR, epidermal growth factor receptor.

** Correlation is significant at the 0.01 level (two-tailed).

* Correlation is significant at the 0.05 level (two-tailed).

Supplementary Table S3. Spearman correlation analysis in the LUSC cohort.

		ADAM10	Pack-Years	Age	TP53	CDKN2A	SOX2	PIK3CA	NOTCH1
ADAM10	ρ	1	.019	.122**	-.040	.020	-.035	.269**	.047
	P		.694	.007	.378	.654	.439	<.001	.301
Pack-Years	ρ	.019	1	.016	-.007	-.001	.012	.023	.016
	P	.694		.740	.880	.979	.813	.645	.740
Age	P	.122**	.016	1	-.009	.008	-.090*	-.054	-.017
	P	.007	.740		.836	.858	.046	.234	.706
TP53	ρ	-.040	-.007	-.009	1	-.021	.041	.052	.124**
	P	.378	.880	.836		.643	.366	.252	.006
CDKN2A	ρ	.020	-.001	.008	-.021	1	.029	-.011	-.025
	P	.654	.979	.858	.643		.526	.805	.582
SOX2	ρ	-.035	.012	-.090*	.041	.029	1	.612**	.137**
	P	.439	.813	.046	.366	.526		<.001	.002
PIK3CA	ρ	.269**	.023	-.054	.052	-.011	.612**	1	.136**
	P	<.001	.645	.234	.252	.805	<.001		.003
NOTCH1	ρ	.047	.078	-.017	.124**	-.025	.137**	.136**	1
	P	.301	.113	.706	.006	.582	.002	.003	

LUSC, lung squamous cell carcinoma; ADAM10, a disintegrin and metalloproteinase 10; TP53, tumor protein p53; CDKN2A, cyclin-dependent kinase inhibitor 2A; SOX2, SRY-box transcription factor 2; PIK3CA, phosphatidylinositol-4,5-bisphosphate 3-kinase catalytic subunit alpha; NOTCH1, notch receptor 1.

** Correlation is significant at the 0.01 level (two-tailed).

* Correlation is significant at the 0.05 level (two-tailed).

Supplementary Table S4. Comparison of ADAM10 expression according to KRAS and EGFR mutation status

Gene	WT: Median (IQR)	Mutant: Median (IQR)	Mann–Whitney P
EGFR	1657.82 (1196.67–2158.29)	1523.99 (1221.28–1944.91)	0.438
KRAS	1657.68 (1198.66–2095.51)	1640.51 (1194.10–2180.25)	0.986
BRAF	1657.95 (1221.28–2120.57)	1313.72 (1000.86–2053.76)	0.178

Data are presented as median (interquartile range [IQR]). P values were calculated using the Mann–Whitney U test.

Supplementary Table S5. Univariate and multivariate Cox proportional hazards regression analyses of overall survival in patients with LUAD.

Variable	Univariate HR(95% CI)	P value	Multivariate HR(95% CI)	P value
Age(>65 vs ≤65)	1.190(0.886 – 1.598)	0.248	1.307(0.836 – 2.043)	0.241
Gender(Male vs Female)	0.889(0.663 – 1.192)	0.432	0.988(0.634 – 1.539)	0.957
Smoking(Yes vs No)	0.907(0.666 – 1.236)	0.536	0.782(0.485 – 1.259)	0.311
Stage(III-IV vs I-II)	2.462(1.797 – 3.371)	<0.001	2.323(1.483 – 3.640)	<0.001
EGFR mutation(Mutant vs Wt)	1.423(0.786 – 2.577)	0.244	1.315(0.692 – 2.499)	0.403
ADAM10(High vs Low)	1.479(1.098 – 1.994)	0.010	1.338(0.858 – 2.088)	0.199

LUAD, lung adenocarcinoma; HR, hazard ratio; CI, confidence interval; EGFR, epidermal growth factor receptor; Wt, wild type.

Supplementary Table S6. Univariate and multivariate Cox proportional hazards regression analyses of overall survival in patients with LUSC.

Variable	Univariate HR(95% CI)	P value	Multivariate HR(95% CI)	P value
Age(>65 vs ≤65)	1.270(0.954 – 1.693)	0.102	1.294(0.890 – 1.882)	0.177
Gender(Male vs Female)	0.831(0.603 – 1.145)	0.258	1.033(0.698 – 1.529)	0.871
Smoking(Yes vs No)	0.971(0.652 – 1.445)	0.883	0.783(0.452 – 1.358)	0.384
Stage(III-IV vs I-II)	1.526(1.108 – 2.103)	0.010	1.671(1.104 – 2.530)	0.015
EGFR mutation(Mutant vs Wt)	1.072(0.523 – 2.197)	0.850	1.128(0.544 – 2.336)	0.747
ADAM10(High vs Low)	1.308(0.997 – 1.715)	0.053	1.312(0.925 – 1.862)	0.128

LUSC, lung squamous cell carcinoma; HR, hazard ratio; CI, confidence interval; EGFR, epidermal growth factor receptor; Wt, wild type.
